# Effects of B_2_O_3_ on Melting Characteristics and Temperature-Dependent Viscosity of High-Basicity CaO–SiO_2_–FeO_x_–MgO Slag

**DOI:** 10.3390/ma13051214

**Published:** 2020-03-08

**Authors:** Junkai Chong, Yingying Shen, Peng Yang, Jianke Tian, Wenjuan Zhang, Xingchang Tang, Xueyan Du

**Affiliations:** School of Materials Science and Engineering, State Key Laboratory of Advanced Processing and Recycling of Nonferrous Metals, Lanzhou University of Technology, Lanzhou 730050, China; chongjunkai@163.com (J.C.); yangpeng02101@163.com (P.Y.); tianjianke@126.com (J.T.); wenjuanzhang86@163.com (W.Z.); tangxingchanglut@163.com (X.T.)

**Keywords:** melting characteristics, viscosity, B_2_O_3_, break temperature, CaO–SiO_2_–FeO_x_–MgO–B_2_O_3_ slag system

## Abstract

In order to reduce the amount of fluorite during the steelmaking process for environmental protection, it is essential to investigate the fluorine-free slag system. Thus, high-basicity CaO–SiO_2_–FeO_x_–MgO slag with B_2_O_3_ content from 0% to 15% was designed, and its melting characteristics and viscosity were investigated. The influence of B_2_O_3_ content on the phase diagram of the slag system was calculated using FactSage 7.3, and the break temperature was determined from the curves of temperature-dependent viscosity. The results show that, with the increase in B_2_O_3_ content, the melting characteristics of the CaO–SiO_2_–FeO_x_–MgO/B_2_O_3_ slag system, including liquidus temperature, flow temperature, softening temperature, and hemispheric temperature, all decreased; the main phase of the slag system transformed from Ca_2_SiO_4_ into borosilicate, and finally into borate; the viscous flow activation energy reduced from 690 kJ to 130 kJ; the break temperature reduced from 1590 °C to 1160 °C. Furthermore, the melting characteristics and the break temperature of the slag system with 5% and 8% B_2_O_3_ content were found to be the closest to the values of fluorine-containing steel slag.

## 1. Introduction

World crude steel production was 1816.61 million tons in 2018, 70.64% of which was produced using an oxygen converter [1]. As an important physical property of slag, melting characteristics and viscosity have great influence on element diffusion, the reaction between slag and steel, gas escape, heat transfer, metal loss, and lining life. Fluorite is often added as a slag melting agent in order to reduce the melting characteristics and viscosity of slag in the process of converter steelmaking. If the fluorite usage is 2–3 kg/t in general, it can consume metallurgical grade fluorite of 1.64–2.46 million tons, based on the crude steel capacity of 820.20 million tons produced via converter in China in 2018 [2].

The main component in fluorite is CaF_2_, and its utilization can cause lots of problems, such as environmental pollution, health hazards, and erosion of furnace linings. In addition, China no longer issues new “fluorite mining licenses” as of 2003 in order to protect fluorite resources. Furthermore, fluorite resources were officially listed as national strategic mineral resources in the “National Mineral Resources Planning (2016–2020)” [3] that was issued by the Ministry of Natural Resources in 2016. It is, thus, imperative to find a new fluorine-free alternative that can maintain almost the same physicochemical properties of the fluorine-containing slag system. Many scholars studied the substitution of fluorite in different slag systems, such as Li_2_O, B_2_O_3_, BaO, MgO, Na_2_O, etc. [4,5,6,7]. The influence of different additives on the melting characteristics and viscosity of slag systems was widely studied. In the CaO–SiO_2_–MgO–Al_2_O_3_ system, the ability to reduce melting characteristics follows the order Li_2_O > Na_2_O > B_2_O_3_, while MgO can only increase the melting characteristics [8]. The order of reducing viscosity is Li_2_O > B_2_O_3_ > BaO > MgO > Na_2_O > K_2_O in the CaO–SiO_2_–Al_2_O_3_–Na_2_O–K_2_O system (mass percentage) [9]. These studies provide the possibility to design a new fluorine-free converter slag system. 

B_2_O_3_ as a good alternative to fluorite due to its similar physical and chemical properties, and it was introduced into many kinds of slags to adjust their viscosity and melting characteristics. It was reported that the addition of B_2_O_3_ not only supplies [BO_3_]-trihedral units into the silicate network, resulting in reducing the symmetry and uniformity of the network structure, but also decreases the break temperature of mold flux and improves the superheat degree, leading to a reduction of the mold flux viscosity [10,11,12,13]. However, research focusing on the effects of B_2_O_3_ on the melting behavior, viscosity, desulfurization, and dephosphorization of the new slag is scarcely reported. This article, thus, discusses the effects of B_2_O_3_ on the melting behavior and viscosity of the designed slag system.

## 2. Methodology and Experiment

The composition of converter steel slag from Gansu JISCO Iron and Steel Co., Ltd. (Jiuquan, China) was determined using the ICP-OES method (Inductively coupled plasma optical emission spectrometry, Thermo Scientific, iCAP 7400 ICP-OES), as shown in Table 1. According to the main components of the slag, the experimental slag system was simplified as a CaO–SiO_2_–FeO_x_–MgO system.

### 2.1. Preparation of Pre-Melted Slag

The samples were prepared using reagent-grade SiO_2_, CaO, MgO, Fe_2_O_3_, Fe, and B_2_O_3_ powders (analytically pure, Sinopharm Chemical Reagent Co., Ltd., Shanghai, China). CaO was calcined at 1273 K (1000 °C) for 10 h in a muffle furnace to decompose any carbonate and hydroxide present before being used. The FeO_x_ was prepared via heating Fe and Fe_2_O_3_ at 900 °C for 2 h under 100 mL/min of Ar gas in a high-temperature tube furnace (molar ratio 1:1). As B_2_O_3_ content increased from 0% to 15%, the mass percentage of MgO remained constant, approaching that of actual steelmaking slag, i.e., MgO/(CaO + SiO_2_ + FeO + MgO) (g/g) = 0.0685. Each reagent was accurately weighed according to the compositions shown in Table 2 and mixed entirely in an agate mortar. The well-mixed reagents were pressed into a cylindrical mold with a diameter of 30 mm and a height of 10 mm under a pressure of 20 MPa. Then, the compressed samples were placed into a vacuum arc furnace (NMS-DRX II, Chengdu Zhongke New Material Technology Engineering Co., Ltd., Chengdu, China) and heated with a current of 300 A for 100 s to ensure that the samples melted in the argon atmosphere. The samples were inverted, and the above smelting process was repeated four times to ensure the homogeneity of the slag samples. Finally, the obtained samples were crushed using a 200-mesh sieve (0.074 mm).

### 2.2. Calculation Method Using FactSage

FactSage 7.3 software (version 7.3, Montreal, Canada) was used to predict the melting characteristics and phase diagrams for the slag systems. The influence of B_2_O_3_ content on the phase diagram and melting characteristics of the CaO–SiO_2_–FeO_x_–MgO/B_2_O_3_ system was calculated using the Phase Diagram and Equilib modules in FactSage 7.3, respectively.

### 2.3. Determination of Melting Characteristics 

**Figure 1:**
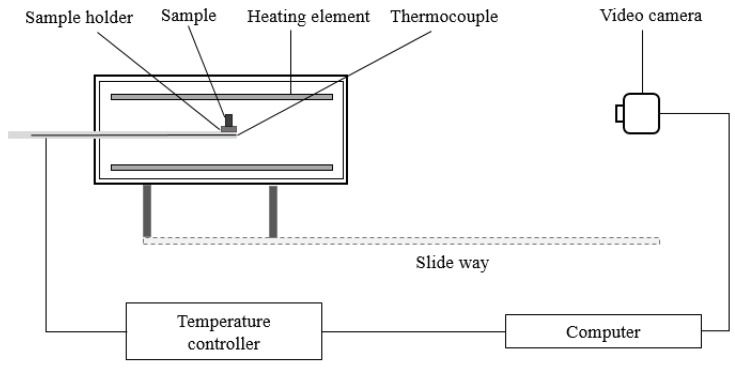
Schematic diagram of LZ-III slag tester used for determination of melting characteristics.

The sample melting characteristics were measured using a hot-stage microscopy method. The experimental equipment (LZ-III slag MCT tester, Northeast University, Shenyang, China) comprised a high-temperature furnace with accurate high-temperature control, as well as a video image-recording and processing system, as shown in Figure 1.

**Figure 2:**
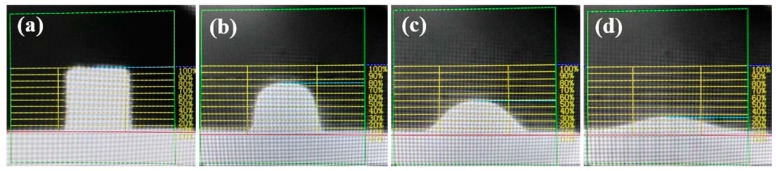
The height changes during the slag melting process: (**a**) the original height; (**b**) the corresponding height of softening temperature; (**c**) the corresponding height of hemispheric temperature; (**d**) the corresponding height of flow temperature.

The samples were pressed into a cylinder with a diameter of 3 mm and a height of 3 mm, and then put into the furnace and heated with a controllable rate of 15 °C/min. At the same time, the change in sample height was observed through the video image-recording system, as shown in Figure 2. Melting characteristics, including the softening temperature (*T*_s_), hemisphere temperature (*T*_h_), and “fluidity temperature (*T*_f_), were defined according to the sample heights of 75%, 50%, and 25%, compared with the original height, respectively [14]. These three temperatures are generally used to characterize the melting trajectory of flux in industrial applications. For instance, the hemispherical temperature is referred to as the melting characteristic of mold flux [15]. The same equipment was described in detailed in a previous paper [16].

### 2.4. Viscosity Measurements

**Figure 3:**
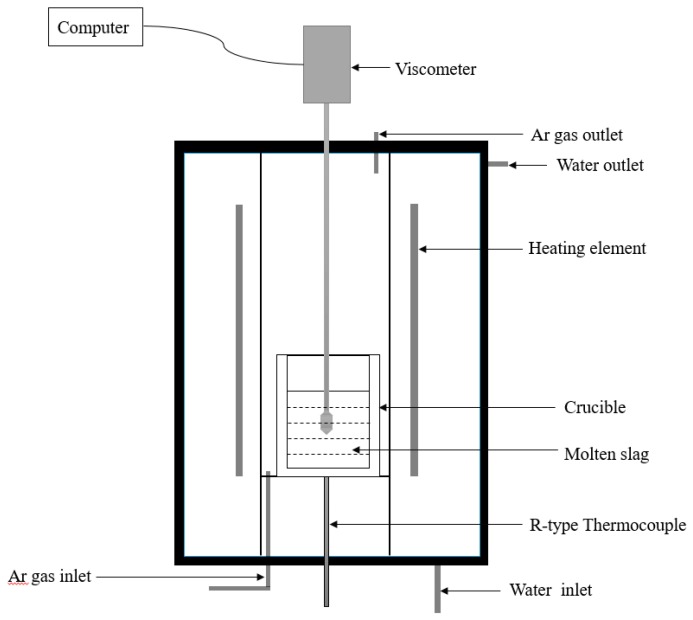
Schematic diagram of the RTW-16 High-Temperature Melt Property Tester.

Viscosity measurements were carried out using a rotary viscometer (RTW-16 High-Temperature Melt Property Tester, Northeast University, Shenyang, China), as shown in Figure 3.

The operation process was as follows: (1) 120 g of obtained slag was pressed into a cylinder with a diameter of 30 mm and a height of 10 mm, at a pressure of 20 MPa, using a tableting machine; (2) the pressed samples were placed into an MgO crucible with a diameter of 40 mm and a height of 120 mm, before heating up to 1600 °C with a heating rate of 3 °C/min, held for 2 h in the RTW-16 High Temperature Melt Property Tester; (3) the corundum rotor was immersed into the slag melt, keeping a distance of 10mm from the bottom of the MgO crucible. The viscosity was measured at the rotation rate of 200 rpm, and the values were recorded during the cooling process with a cooling rate of 3 K/min. High-purity argon gas (99.99%, 1.5 L/min) was introduced as the protective gas during the measurement process. The measurement was terminated when the viscosity value was close to 3.5 Pa·s. Castor oil was used to calibrate the instrument [16].

## 3. Results and Discussion

### 3.1. Effects of B_2_O_3_ on Phase and MCT of CaO–SiO_2_–FeO_x_–MgO System

**Figure 4:**
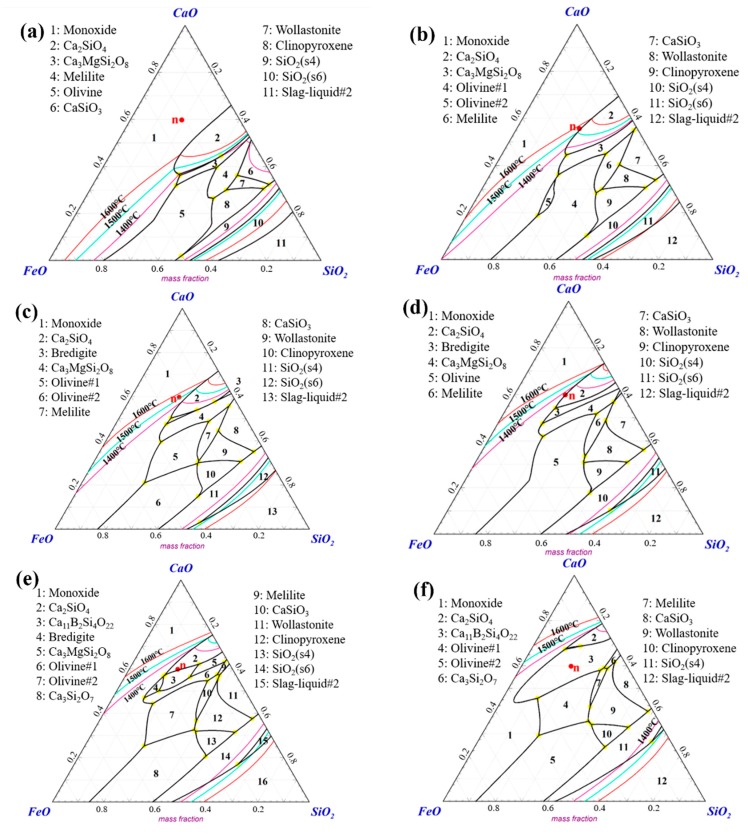
Calculated phase diagrams of CaO–SiO_2_–FeO–MgO system with different content of B_2_O_3_, drawn using FactSage: (**a**) B_2_O_3_ = 0%; (**b**) B_2_O_3_ = 5%; (**c**) B_2_O_3_ = 8%; (**d**) B_2_O_3_ = 10%; (**e**) B_2_O_3_ = 12%; (**f**) B_2_O_3_ = 15%.

Figure 4 shows the phase diagrams of the CaO–SiO_2_–FeO–MgO/B_2_O_3_ system with different contents of B_2_O_3_, drawn using FactSage software. It can be seen that the composition point was located in the monoxide phase region (i.e., primary phase region) without B_2_O_3_ addition. With the B_2_O_3_ content increasing, the primary phase transformed into Ca_2_SiO_4_, and then reached the Ca_11_B_2_Si_4_O_22_ phase region. Furthermore, the liquidus temperature changed from an initial value higher than 1600 °C down to less than 1400 °C.

**Figure 5:**
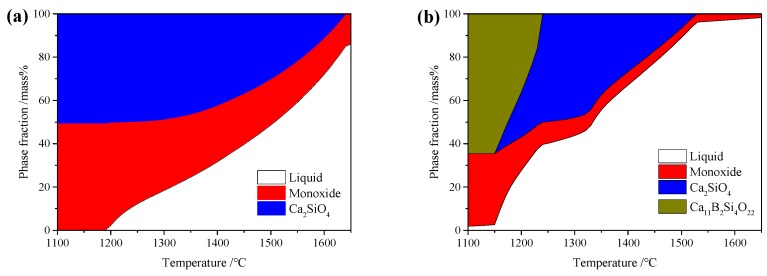

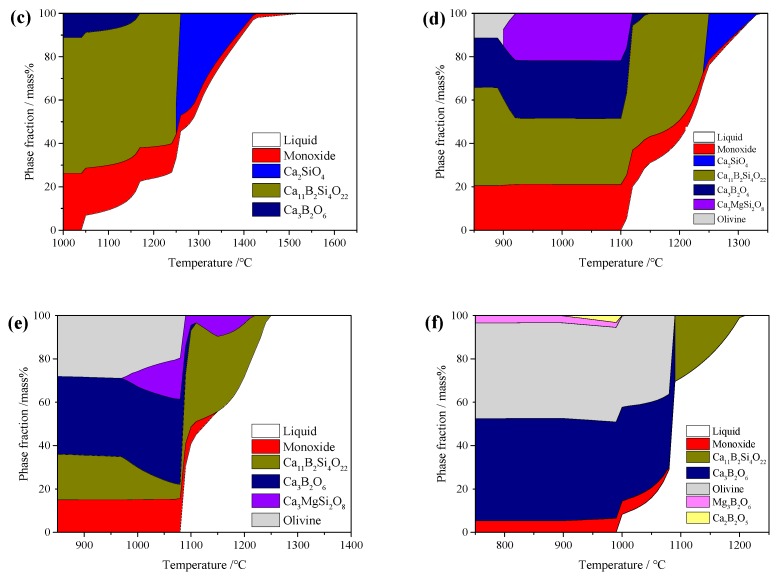
Calculated equilibrium phase fractions of CaO–SiO_2_–FeO–MgO/B_2_O_3_ system, drawn using FactSage: (**a**) B_2_O_3_ = 0%; (**b**) B_2_O_3_ = 5%; (**c**) B_2_O_3_ = 8%; (**d**) B_2_O_3_ = 10%; (**e**) B_2_O_3_ = 12%; (**f**) B_2_O_3_ = 15%.

Figure 5 shows the equilibrium phase fractions of the CaO–SiO_2_–FeO–MgO/B_2_O_3_ system calculated using FactSage 7.3. It can be seen that the main components in the slag system were Ca_2_SiO_4_ and monoxide without B_2_O_3_ addition, and then Ca_11_B_2_Si_4_O_22_ and Ca_3_B_2_O_6_ occurred at 5% B_2_O_3_ content and 8% B_2_O_3_ content, respectively. When the B_2_O_3_ content continued to increase to 12%, the main phases in the slag system were Ca_11_B_2_Si_4_O_22_ and Ca_3_B_2_O_6_, as well as olivine. Finally, the main boron-containing phases in the slag system became Ca_3_B_2_O_6_ and olivine at 15% B_2_O_3_ content. Moreover, the liquidus temperature decreased from an initial value of1650 °C down to 1210 °C with the increase in B_2_O_3_ content.

**Figure 6:**
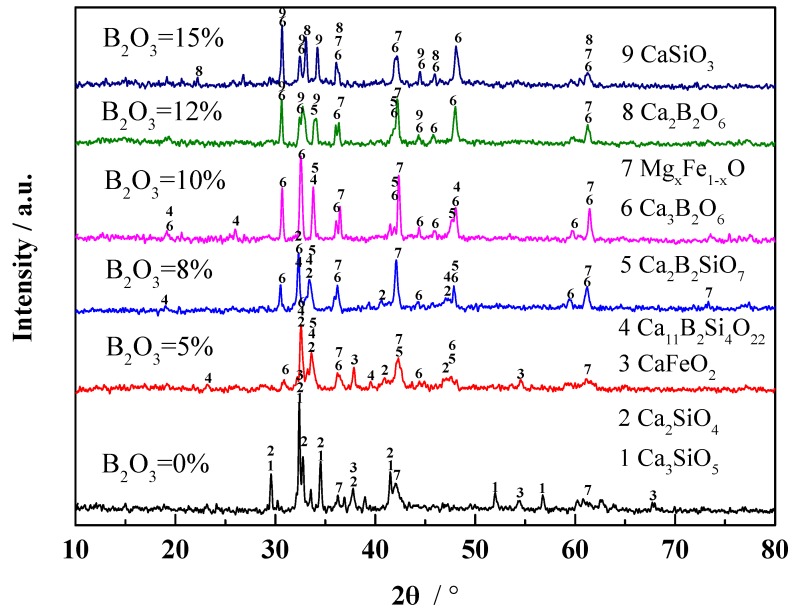
X-ray diffraction (XRD) patterns of CaO–SiO_2_–FeO_x_–MgO/B_2_O_3_ systems with different B_2_O_3_ content.

In order to confirm the accuracy of FactSage predictions, X-ray diffraction (XRD) analysis was performed for all samples, as shown in Figure 6. It was found that the main phases of the slag system were Ca_2_SiO_4_, Ca_3_SiO_5_, and monoxide (CaFeO_2_, Mg_x_Fe_1−x_O) without B_2_O_3_ addition. When the B_2_O_3_ content was 5%, Ca_11_Si_4_B_2_O_22_ and Ca_2_B_2_SiO_7_ occurred. When the B_2_O_3_ content was increased to 8%, Ca_3_B_2_O_6_ formed. When the B_2_O_3_ content was increased to 12% and 15%, the main phases changed to Ca_3_B_2_O_6_ and CaSiO_3_. Most of these phases were identical to the results of Figure 5, implying that the prediction of FactSage was convincing. It was also found that the slag structure initially became complicated and then gradually changed into a simple structure with the phase transformation caused by the addition of B_2_O_3_ [17].

**Figure 7:**
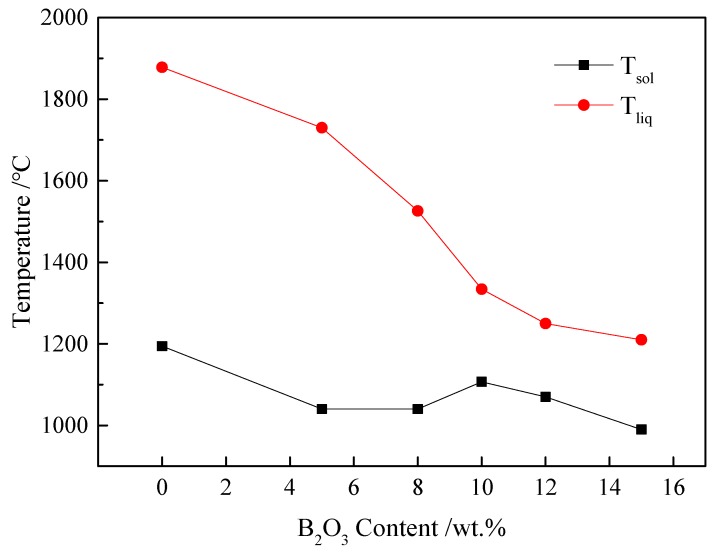
Variation in *T*_liq_ and *T*_sol_ of CaO–SiO_2_–FeO–MgO/B_2_O_3_ system with B_2_O_3_ content calculated using FactSage.

Generally, the liquidus temperature (*T*_liq_) and solidus temperature (*T*_sol_) are defined as the maximum and minimum temperatures in the coexistence region of liquid and solid phases, respectively [10]. According to the equilibrium phase fractions calculated using FactSage (shown in Figure 5), *T*_liq_ and *T*_sol_ of the CaO–SiO_2_–FeO–MgO/B_2_O_3_ system could be calculated with different B_2_O_3_ content, as shown in Figure 7. It can be seen that, as the B_2_O_3_ content increased, *T*_liq_ decreased continuously, while *T*_sol_ decreased firstly, then increased slightly, and finally decreased.

**Figure 8:**
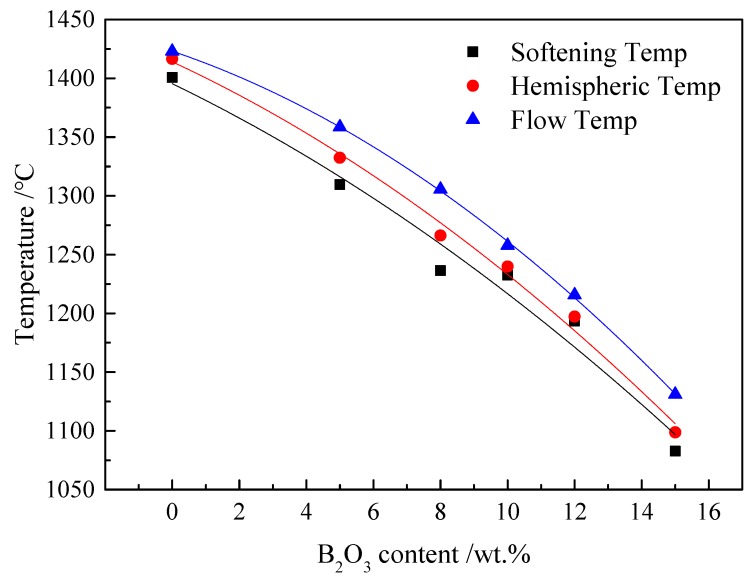
Variation in melting characteristics (*T*_s_, *T*_h_, and *T*_f_) of CaO–SiO_2_–FeO_x_–MgO/B_2_O_3_ system with B_2_O_3_ content_._

Melting characteristics (*T*_s_, *T*_h_, and *T*_f_) are closely related to the formation and melting of solid phases in the flux melting process [14]. Figure 8 shows the effects of B_2_O_3_ addition on the melting characteristics (*T*_s_, *T*_h_, and *T*_f_) of the CaO–SiO_2_–FeO_x_–MgO/B_2_O_3_ system. It was found that, with B_2_O_3_ content increasing, *T*_s_, *T*_h_, and *T*_f_ decreased from 1400.60 °C to 1082.80 °C, 1414.60 °C to 1098.80 °C, and 1423.00 °C to 1131.00 °C, respectively. Compared with the melting characteristics of actual converter steel slag (*T*_f_ = 1420.00 °C, *T*_h_ = 1414.00 °C, *T*_s_ = 1405.00 °C), the values of the CaO–SiO_2_–FeO_x_–MgO/B_2_O_3_ system with 5% B_2_O_3_ were found to be the closest to that of actual fluorine-containing steel slag, indicating that this slag system could be used as a candidate for steelmaking applications.

### 3.2. Effects of B_2_O_3_ on Break Temperature and Apparent Activation Energy of CaO–SiO_2_–FeO_x_–MgO System

Break temperature [18,19,20,21] is defined by the intersection of the two tangents of the linearized curve branches within a logarithmic plot of viscosity versus the reciprocal Kelvin temperature (K^−1^), which refers to the temperature at which the fluid undergoes non-equilibrium solidification during the cooling process, and suddenly changes from a Newtonian fluid to non-Newtonian fluid. The break temperature (*T*_br_) is shown by the abrupt change in viscosity, which separates the fully liquid region from the solid–liquid coexisting region, and it represents the point at which solids are first precipitated in the melt. The same expression was also expressed in a large number of fly-ash-related articles [22,23], but this temperature was called the critical viscosity temperature.

**Figure 9:**
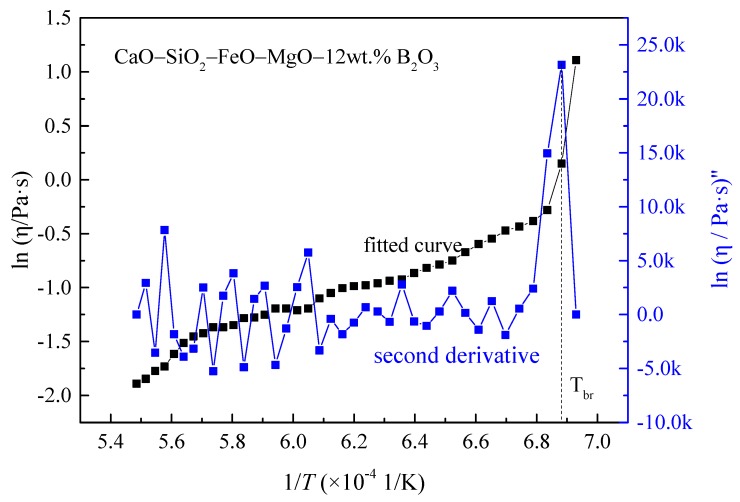
The natural logarithm of viscosity as a function of 1/*T* for the CaO–SiO_2_–FeO_x_–MgO slag system.

**Figure 10:**
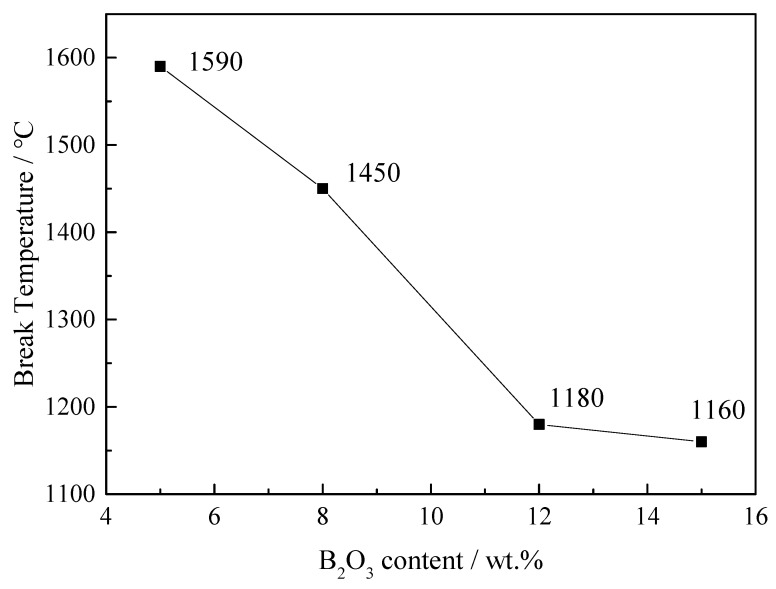
Break temperature of slag systems with different content of B_2_O_3_.

The break temperature of the CaO–SiO_2_–FeO_x_–MgO/B_2_O_3_ slag system was accordingly investigated based on the temperature-dependent viscosity. The curve of ln η vs. 1/*T* of the 12% B_2_O_3_ system is plotted as an example in Figure 9. Second-order polynomials were determined from three consecutive pairs of log η versus 1/*T* values, from which the second derivative of log η at the center point was calculated, where the maximum absolute value was identified the break temperature [21]. Break temperature was, thus, calculated, and the results are plotted in Figure 10. It can be seen that, with the content of B_2_O_3_ increasing, the break temperature decreased significantly. The addition of B_2_O_3_ can inhibit crystallization, and it would also combine with other oxides in the slag to form low-melting-point eutectics, leading to a reduction in break temperature [20,24]. 

**Figure 11:**
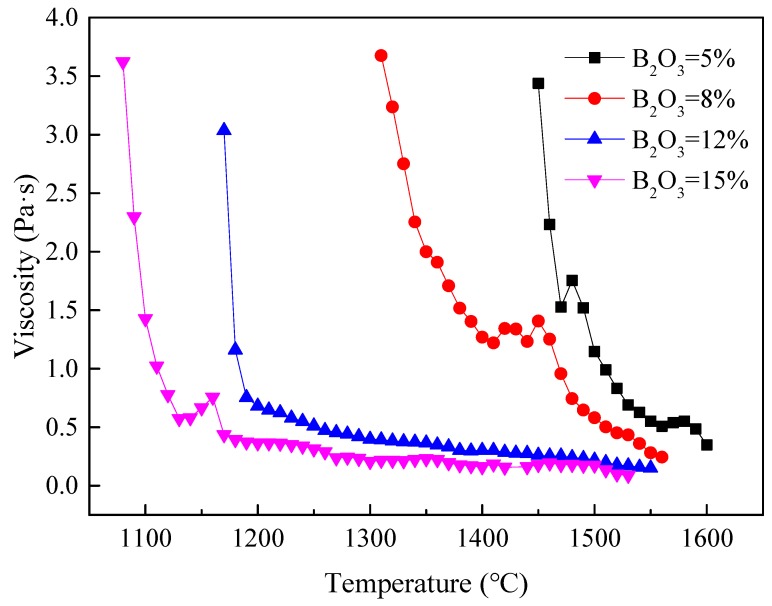
Viscosity of slag systems with different content of B_2_O_3_.

Figure 11 shows the viscosity–temperature curves of the B_2_O_3_-containing slag system. The viscosity values were captured at an interval of 10 °C during the cooling process. It can be seen that, with the increase in B_2_O_3_ content, the viscosities decreased sharply, and the corresponding break temperature also reduced. Meanwhile, at the same temperature, the viscosities decreased with the increase in B_2_O_3_ content, which could be attributed to the slag structure and liquid fraction. In a complete molten state, the viscosity is mainly determined by the melt structure, where simpler [BO_3_]-trihedral units replace complicated silicate network units, resulting in the viscosity decreasing [11,25,26]. In the coexistence state of liquid and solid phases, the viscosity is mainly affected by the liquid fraction; according to the Roscoe–Einstein equation [27], the viscosity is mainly determined by the solid content in the system, also resulting in the viscosity decreasing. 

**Figure 12:**
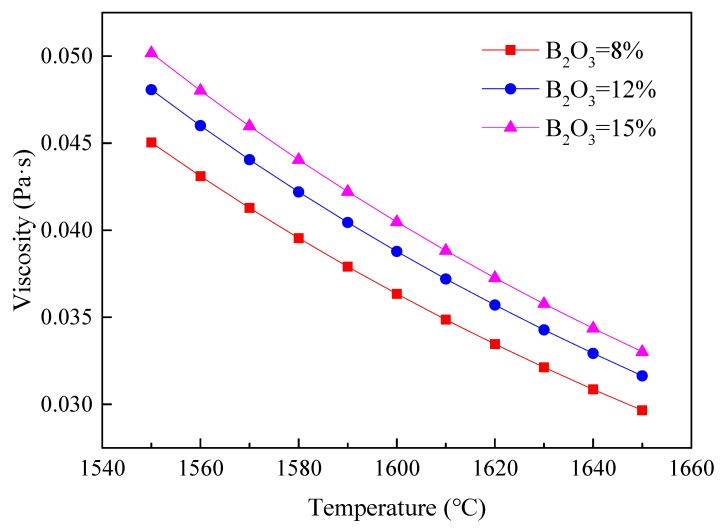
Viscosity of slag systems with different contents of B_2_O_3_ in liquid phase calculated using FactSage.

It should be mentioned that the viscosity of the liquid in the slag system was calculated using FactSage software, as shown in Figure 12. It can be found that the viscosity increased with the rise in B_2_O_3_ content, in contrast to the experimental results shown in Figure 11. As reported in the literature [10,17], this interesting phenomenon is worthy of investigation to understand the difference between FactSage calculation results and experimental measurement results.

**Figure 13:**
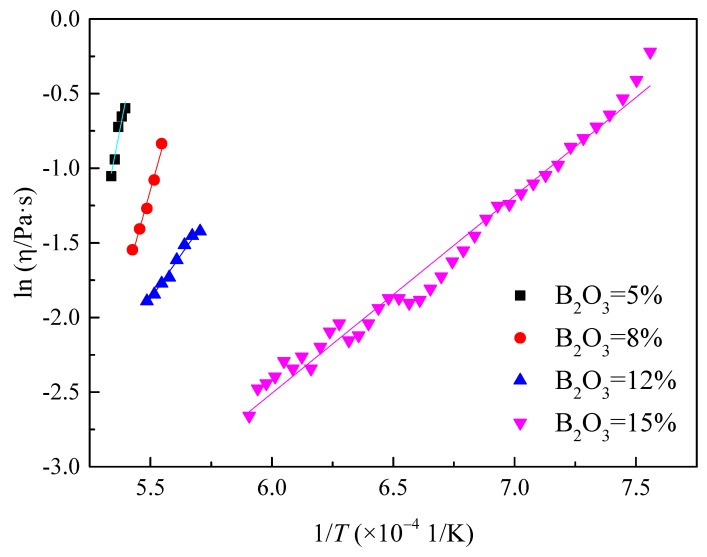
Relationship between ln *η* and 1/*T* with different B_2_O_3_ content according to the Arrhenius equation.

**Figure 14:**
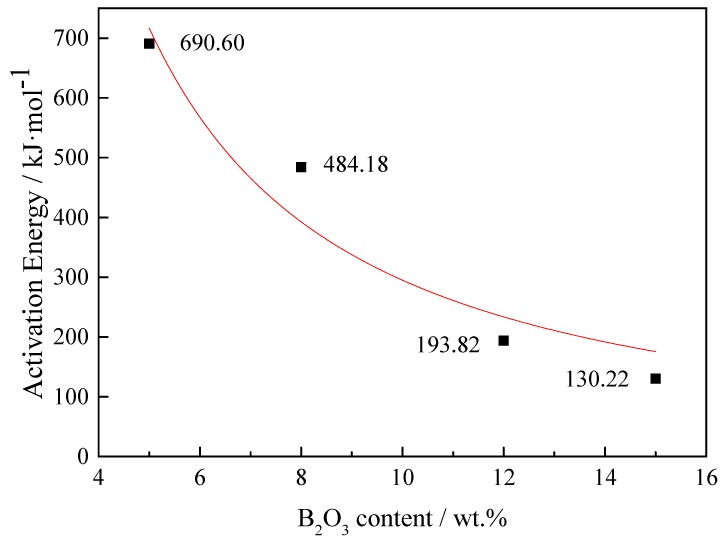
Variation in apparent activation energy of viscous flow with B_2_O_3_ content.

The viscosity of borosilicate melts is strongly dependent on the degree of polymerization, which is a function of temperature and composition. Their relationship is generally discussed by taking the activation energy for viscous flow into account. The activation energy for viscous flow of silicate melts can be calculated using the following Arrhenius equation [28]:

(1) $$\eta =\eta_{0}\exp\frac{E\eta }{RT}$$

where *η*, η_0_, *E*η, R, and *T* are the viscosity, a pre-exponent constant, the activation energy, the gas constant, and absolute temperature, respectively. Hence, it is possible to calculate *E*η above the break temperature according to the plots of ln *η* vs. 1/*T*, as shown in Figure 13. Eη represents the energy barrier for viscous flow, the variations of which imply the structure changes of the molten slag and which further reflect the transformation of the flow units in the slag [10]. The activation energy with different B_2_O_3_ content was then calculated, as shown in Figure 14. It can be seen that the activation energy gradually decreased from 690 kJ to 130 kJ with increasing B_2_O_3_ content.

This can be attributed to the change in slag structure. With the B_2_O_3_ content increasing, two-dimensional (2D) triangular [BO_3_] units occur in the slag system, gradually becoming the dominant structure associated with B–O arrangements, favoring slag flowability and resulting in an apparent reduction in activation energy [17].

## 4. Conclusions

(1) With the increase in B_2_O_3_ content, the melting characteristics including liquidus temperature, softening temperature, and hemispheric temperature of the CaO–SiO_2_–FeO_x_–MgO/B_2_O_3_ slag system all decreased. The melting characteristics of the slag system with 5% B_2_O_3_ were found to be the closest to that of actual fluorine-containing steel slag, indicating that this slag system could be used as a candidate for steelmaking applications.

(2) With the increase in B_2_O_3_ content, borosilicate and borate occurred in the CaO–SiO_2_–FeO_x_–MgO/B_2_O_3_ slag system, resulting in the slag structure initially becoming complicated before gradually changing into a simple structure.

(3) With the increase in B_2_O_3_ content, the break temperature was reduced from 1590 °C to 1160 °C, and the viscous flow activation energy was reduced from 690 kJ to 130 kJ.

## Figures and Tables

**Table 1:** Compositions of steel slag (mass fraction, %).

CaO	FeO_x_	SiO_2_	MgO	Al_2_O_3_	Other
50.04	17.87	15.86	6.17	4.08	5.98

**Table 2:** Compositions of the samples (mass fraction, %).

Sample	CaO	SiO_2_	FeO_x_	MgO	B_2_O_3_
A1	55.64	17.63	19.87	6.86	0.00
A2	52.86	16.75	18.88	6.51	5.00
A3	51.19	16.22	18.28	6.31	8.00
A4	50.07	15.87	17.89	6.17	10.00
A5	48.96	15.52	17.49	6.03	12.00
A6	47.29	14.99	16.89	5.83	15.00
